# Modulation of reaching by spatial attention

**DOI:** 10.3389/fnint.2024.1393690

**Published:** 2024-05-15

**Authors:** Rossella Breveglieri, Riccardo Brandolani, Stefano Diomedi, Markus Lappe, Claudio Galletti, Patrizia Fattori

**Affiliations:** ^1^Department of Biomedical and Neuromotor Sciences, University of Bologna, Bologna, Italy; ^2^Center for Neuroscience, University of Camerino, Camerino, Italy; ^3^Department of Psychology, Otto Creutzfeldt Center for Cognitive and Behavioral Neuroscience, University of Münster, Münster, Germany

**Keywords:** covert attention, reaching, Principal Components Analysis, hemispatial effects, right-handed people, left handed people

## Abstract

Attention is needed to perform goal-directed vision-guided movements. We investigated whether the direction of covert attention modulates movement outcomes and dynamics. Right-handed and left-handed volunteers attended to a spatial location while planning a reach toward the same hemifield, the opposite one, or planned a reach without constraining attention. We measured behavioral variables as outcomes of ipsilateral and contralateral reaching and the tangling of behavioral trajectories obtained through principal component analysis as a measure of the dynamics of motor control. We found that the direction of covert attention had significant effects on the dynamics of motor control, specifically during contralateral reaching. Data suggest that motor control was more feedback-driven when attention was directed leftward than when attention was directed rightward or when it was not constrained, irrespectively of handedness. These results may help to better understand the neural bases of asymmetrical neurological diseases like hemispatial neglect.

## Introduction

When we manually interact with an object that is located either on the same side as the hand used (ipsilateral reaching) or on the opposite side (contralateral reaching), hemispatial effects, i.e., hemispatial advantages in behavioral measures during ipsilateral reaching, have been reported in several studies (Berlucchi et al., 1971; Van Der Staak, 1975; Prablanc et al., 1979; Di Stefano et al., 1980; Bashore, 1981; Fisk and Goodale, 1985; Carson et al., 1990, 1992, 1993; Marzi et al., 1991; Chua et al., 1992; Hoptman and Davidson, 1994; Ingum and Bjørklund, 1994; Carey et al., 1996; Levin, 1996). For example, ipsilateral reaching is faster than contralateral reaching and has a higher peak velocity that is likely due to biomechanical constraints (Carey et al., 1996). It has been suggested that the allocation of attention could be related to these effects, given that right-handers may attend more frequently to the right space and/or the right hand (Honda, 1984; Peters, 1987; Bradshaw et al., 1989; Fagot et al., 1994; Rizzolatti et al., 1995). In line with this view, the ability to direct attention toward the targets of action is essential for our interactions with objects. Previous research on the interplay between attention and movement has suggested that attention influences motor control: when participants are instructed to focus on the goal of a task, their neuromuscular coordination improved compared to when they were instructed to focus on their internal body mechanics (Lohse et al., 2012), or when no attentional instructions are given (Wulf et al., 2007). Attention also affects properties of the movement itself, such as muscle recruitment (Vance et al., 2004; Zachry et al., 2005; Lohse and Sherwood, 2012), energetic cost (Schücker et al., 2009), and movement performance in terms of lower absolute error, preparation time and earlier muscle recruitment when focusing externally than internally during a dart throwing movement (Lohse et al., 2010). Moreover, attention reduces errors in the movement outcome (Lohse et al., 2010), and this suggests that it adjusts motor control by helping to determine a regulatory strategy for the motor system (Lohse et al., 2014). However, despite the huge amount of data indicating that attention affects motor actions, the role of attention in motor control is far from being fully established. Most studies have been limited to the evaluation of its effects on motor outcomes (e.g., accuracy, balance, speed), but limited research has been devoted to investigating the impact of attention on the kinematic and dynamic properties of movement, leaving this as an open question.

Attention-related hemispheric asymmetries have been observed in several studies. Healthy participants tend to place a bisection marker to the left of the real midpoint on a horizontal line, a bias called ‘the left visual field bias’ or ‘pseudoneglect’ (Bowers and Heilman, 1980). This bias may be explained by the right hemisphere’s dominance in directing spatial attention (Heilman and Van Den Abell, 1980; Reuter-Lorenz et al., 1990; Benwell et al., 2014), accompanied by the lesser effort required when attending to the left visual field compared to the right (Meyyappan et al., 2023). The dominance of the right hemisphere is also supported by a PET study showing that there are two distinct representations in the right hemisphere for directing attention toward the left or right visual field, but only one representation in the left hemisphere for directing attention mainly into the right visual field. In other words, the right hemisphere could direct attention toward both hemispaces, whereas the left hemisphere only toward the right hemispace (Corbetta et al., 1993). A fMRI study showed that attentional modulation of population receptive field size, an indicator of spatial representation, exhibits asymmetry. Specifically, directing attention to the visual stimulus results in a bilateral spatial representation within the right parietal cortex, in contrast to the left parietal cortex, which remains contralateral (Sheremata and Silver, 2015). The left–right asymmetry is also reflected in the hemispatial neglect following unilateral brain damage. Patients with unilateral right hemisphere lesions show contralesional (left) neglect, whereas in the case of lesions in the left hemisphere the neglect only appears in a few cases (Stone et al., 1992; Bowen et al., 1999; Heilman et al., 2003; Parton et al., 2004; Ten Brink et al., 2017). Finally, people with attention deficit disorder or attention deficit hyperactivity disorder make significantly more errors in the left half of visual targets (Jones et al., 2008), or generally make more left-sided errors (Voeller and Heilman, 1988; García-Sánchez et al., 1997).

In this work, we wanted to investigate the interaction between spatial attention and reaching movement. First, we instructed participants to reach toward ipsilateral or contralateral targets to evaluate the advantages during ipsilateral reaching versus contralateral reaching (Berlucchi et al., 1971; Van Der Staak, 1975; Prablanc et al., 1979; Di Stefano et al., 1980; Bashore, 1981; Fisk and Goodale, 1985; Carson et al., 1990, 1992, 1993; Marzi et al., 1991; Chua et al., 1992; Hoptman and Davidson, 1994; Ingum and Bjørklund, 1994; Carey et al., 1996; Levin, 1996) [the so-called ‘hemispatial effects’ (Carey et al., 1996)]. Then, we studied the influence on hemispatial effects of directing attention leftward or rightward during reach planning and compared them to when attention was not constrained. We used, together with classic kinematic analyses, a new state-of-the-art analysis which evaluates the dynamical system underlying motor control. With this novel approach, we evaluated whether attention directed to the right or to the left visual field during reach planning affects the dynamics of motor control during the execution of ipsilateral and/or contralateral reaching. To see whether brain mechanisms underlying the interactions between attention and movement depend on handedness we also tested right-handers and left-handers subjects, the latter often neglected in studies on arm movement control.

## Materials and methods

### Participants

Thirty-four healthy volunteers (17 males) participated in this study. The participants were classified as right- (*N* = 18, 9 males, aged 23.78+/−3.41, age range 19–30) or left-handed (*N* = 16, 8 males, aged 29.125+/−8.82, age range 21–53) based on the Edinburgh Handedness Inventory (Oldfield, 1971) (scores: mean/SD of right-handed participants = 73.64 +/− 18.72; mean/SD of left-handed participants = −66.66 +/− 12.38), had normal or corrected-to-normal visual acuity in both eyes and were naïve as to the purposes of the experiment. Participants provided written informed consent, and the procedures were approved by the Bioethical Committee at the University of Bologna and were in accordance with the ethical standards of the 2013 Declaration of Helsinki.

### Apparatus and behavioral task

**Figure 1:**
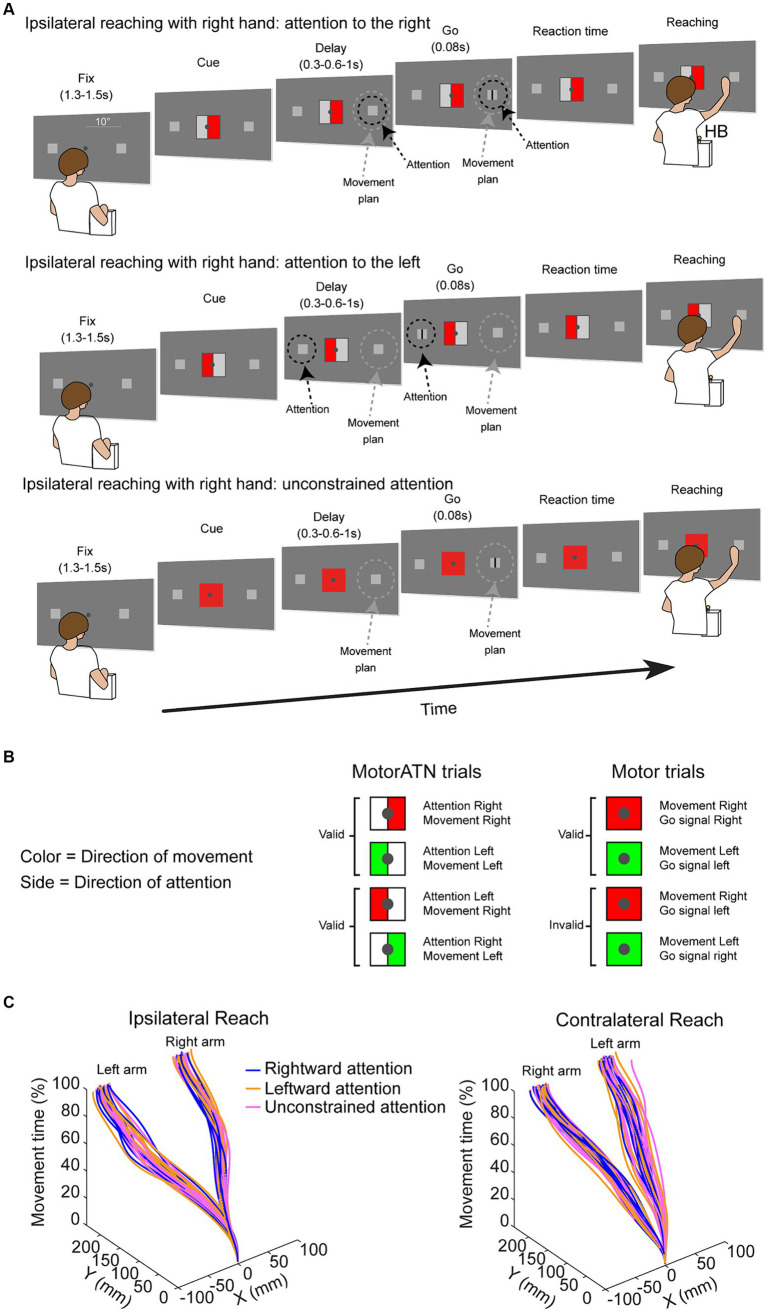
**(A)** Timeline of attention/reaching task. Fix, fixation time; Cue, cue onset; Delay, delay between cue on and go signal; Go, go signal (a small vertical line), Reaction time and Reaching. HB, home button. The Cue is depicted larger than in the real task for the reader’s convenience. Real dimensions are stated in the Methods section. Dashed circles represent the direction of the spotlight of attention (black) and of the motor plan (gray). The timeline is shown for ipsilateral trials in which attention is directed rightward (top), leftward (middle) or is not constrained (bottom). The same timeline also applies to contralateral trials (not shown for conciseness). Trials were performed with either the right or left arm, one per session. **(B)** Types of trials, according to the information received by the central cue: MotorATN trials and Motor trials. The Motor trials could be valid (Go signal in the target cued by the color) or invalid (Go signal in the opposite target). **(C)** Three-dimensional plot of single-trials index trajectories of an exemplary right-handed participant during ipsilateral (left) and contralateral (right) reaching. X, horizontal coordinates; Y, vertical coordinates. Right arm, reaching performed with the right arm; Left arm, reaching performed with the left arm. Colors represent the location of the spotlight of attention during the delay.

We tested the influence of the interactions between the directions of attention and the direction of movement planning on kinematics by using a setup which consisted of a 19-inch touchscreen (ELO IntelliTouch 1939L) set vertically at 43 cm in front of the participants. The screen displayed two targets of the reaching movements performed by the participants (gray squares in Figure 1A, 0.6 cm side, 0.78°, 10° lateral to the fixation point). For stimuli presentation, we used Matlab (Mathworks, USA, R2021b, RRID: SCR_001622) with the Psychophysics toolbox extension (Brainard, 1997). Participants were seated on a comfortable chair in a darkened room, with their head stabilized by a head/chin rest to minimize head movements. In all trials, the reaching movement started with the participant’s hand on a button (home button, HB, Figure 1A) placed on the desk. This button was centrally aligned with both the touchscreen and the trunk of the participants (Figure 1A).

The task was designed to be able to direct covert attention and movement plan independently from each other, as it has been recently demonstrated in a monkey study (Messinger et al., 2021). A cue instructed the direction of attention and the direction of movement plan. The color of the cue told the participant in which direction the movement should be planned, i.e., to which target the subsequent movement must be directed. The side of the cue on which the color was shown informed the participant as to the direction the spotlight of attention should be covertly directed. Each trial started, after an intertrial period of 3 s, with the onset of the fixation point (diameter 0.3 cm, 0.4° of visual angle) in the center of the screen, between the two targets. This indicated that the participant should press and hold down the home button. The two reaching targets were displayed on the touchscreen during the entire duration of each trial. After a fixation period (Fix, Figure 1A) of 1.3–1.5 s (randomly chosen), an endogenous cue (0.6 cm side, 0.76°) appeared around the fixation point, which informed the participants which target they must covertly attend to, and which target should be subsequently reached [motor-attention (MotorATN) trials, Figure 1B], or only which target they should subsequently reach [motor (Motor) trials, Figure 1B]. After a randomly chosen delay period of 0.3–0.6-1 s (stimulus-onset asynchrony, SOA, Delay in Figure 1A), a small vertical line (Go) appeared for 0.08 s in the center of one reaching target. Importantly, we included the 2 trials with SOA = 1 ms in each condition to guarantee that the participants’ attention was directed toward the cued side. We then excluded them from the analyses. After a variable reaction time (Reaction time, Figure 1A) to the detection of the Go signal, participants reached the previously cued target (Reaching, Figure 1A, trajectories of an exemplary right-handed participant, Figure 1C). At movement offset, the targets and fixation point disappeared and another intertrial period started.

In MotorATN trials (Figure 1A, top and intermediate, and 1B, left), the color of the cue informed participants which target location they should subsequently reach (red = reach planning to the right target, green = reach planning to the left target, as in Messinger et al., 2021); the colored side of the cue showed the location of the subsequent Go signal, in order to make participants covertly shift their spotlight of attention toward the cued side (right side colored = attention to the right; left side colored = attention to the left). This task enabled us to test ipsilateral and contralateral reaching, performed with either the right or the left hand, with covert spatial attention directed rightward (Figure 1A, top) or leftward (Figure 1A, middle). Ipsilateral reaching was performed with the right hand toward the right target, or with the left hand toward the left target, whereas contralateral reaching was performed with the right hand toward the left target or with the left hand toward the right target. In MotorATN trials, the Go signal appeared always in the side indicated by the colored side of the cue, thus in the attended target (valid trials).

In Motor trials (Figure 1A, bottom and 1B, right), the central cue was a fully colored square which informed participants as to the location of the movement plan only (same color conventions as in MotorATN trials). In these trials, participants had to plan a reach without any constraints concerning the location toward which endogenous attention must be directed during the delay. To ensure that the attention of the participants was not automatically directed to the location of the movement plan, we inserted valid and invalid trials in equal number (Figure 1B). In valid trials (80% of the total number of trials), the Go signal appeared in the target of the reach plan. Conversely, in invalid trials (20% of the total number of trials) the Go signal appeared in the opposite target. Overall, 8 conditions were tested (4 conditions for MotorATN trials and 4 for Motor trials, Figure 1B). Importantly, endogenous attention was not constrained during movement execution, either in MotorATN trials or in Motor ones.

The task was composed of 2 blocks of 48 trials each per arm (6 trials per condition per block) for a total of 192 trials performed over the same experimental session. Each session lasted for approximately 1 h. In two blocks the task was performed with the dominant arm (right arm for right-handers and left arm for left-handers) and in the other two with the non-dominant arm. We randomized blocks of each arm (dominant, non-dominant) and, in each block, the conditions of MotorATN trials and of Motor trials were also randomized. A 48-trial training block was included at the beginning of the experimental session. To run the task and for data analysis, we used Matlab (Mathworks, USA, R2021b).

### Data acquisition, analysis, and statistics

The kinematics of reaching movements was recorded using a motion tracking system (VICON motion capture system, 6 M cameras, 1,024 × 1,024 pixel resolution) by sampling the position of two markers at a frequency of 100 Hz; markers were attached to the wrist (on the scaphoid bone) and the nail of the index finger (reaching finger/wrist, right when the task was performed with the right hand, left when it was performed with the left hand). Participants were asked to move the hand in a ballistic way (without pauses or interruptions), at a fast but comfortable speed, and as accurately as possible. Reaching onset was determined as the time when the velocity of the markers exceeded 30 mm/s, while offset was set when the velocity fell and remained below 30 mm/s. Reaction time (RT) was defined as the interval between the “Go” signal offset and reaching onset. Movement time was obtained by subtracting the movement onset from the respective movement offset. Eye position was recorded at 1 kHz using an EyeLink 1,000 (SR Research Ltd) eye tracker. Before collecting data from each participant, the equipment was calibrated using a nine-point grid that the participants were asked to fixate steadily.

Given the inherent complexity of the task, there was a potential for participants to inaccurately reach for the wrong target or initiate an incorrect movement trajectory, subsequently correcting it to reach the intended target. To address this problem, we excluded each trial in which the first or the second half of the trajectory exceeded the 2 standard deviations calculated for all the trajectories of that participant. Additionally, we removed trials in which the endpoint of the reach was on the opposite side of the color-cued target. This was done for all trajectories, separately for the 2 targets. We also excluded trials with RTs shorter than 100 ms (Ciavarro et al., 2013) or longer than 1,000 ms (Rizzolatti et al., 1987). Analysis of eye movements was performed offline, but we did not exclude any trials because all the eye traces were within a tolerance window of 3° during the Delay. The total percentage of excluded trials was 8%, considering all the exclusion criteria.

As in previous studies (Vesia et al., 2006, 2008, 2010; Breveglieri et al., 2021), movement accuracy and precision were extracted by endpoints recorded by the touchscreen and derived from the parameters (coordinates: accuracy and area: precision) of 95% confidence ellipses fit to hand position (endpoint) distributions measured at movement offset.

We used Principal Components Analysis (PCA) to reduce the dimensionality of behavioral data and compute a behavioral manifold, similarly to what was performed in other studies (Russo et al., 2018; Perich et al., 2020). First, we filtered the motion tracking data with a fourth order Butterworth filter (Matlab2021 function “butter,” filter type “low”) with 60 Hz as the optimal cut-off frequency to apply to the filter determined by a residual analysis (Winter, 2009). We then z-scored the position $pos\left(t\right)=\left[x_{i}\left(t\right),\;y_{i}\left(t\right),\;z_{i}\left(t\right),\;x_{w}\left(t\right),\;y_{w}\left(t\right),\;z_{w}\left(t\right)\right]$ , velocity $vel\left(t\right)=\left[v_{\mathrm{xi}}\left(t\right),\;v_{yi}\left(t\right),\;v_{zi}\left(t\right),\;v_{xw}\left(t\right),\;v_{yw}\left(t\right),\;v_{zw}\left(t\right)\right]$ and acceleration $acc\left(t\right)=\left[a_{\mathrm{xi}}\left(t\right),\;a_{yi}\left(t\right),\;a_{zi}\left(t\right),\;a_{xw}\left(t\right),\;a_{yw}\left(t\right),\;a_{zw}\left(t\right)\right]$ from the markers placed on the index finger and wrist of the participants (18 total variables). If participants did not have the same number of trials due to individual errors, we homogenized the dataset by adding as many synthetic trials into each condition until there were a total of 10 trials per condition. These synthetic trials were obtained by interpolating each individual trial of each condition to the average length of that condition. The interpolated trials were then averaged to obtain an artificial trial for each condition that averaged the original trials in both content and length. Then, for each subject we obtained a matrix of 18xT (T is the length of all concatenated trials) by concatenating the 18-dimensional vector [pos (t), vel (t), acc (t)] of each trial. We then applied PCA to this set of 18 variables for each subject separately and selected the first 8 Principal Components (PCs) for subsequent analysis, to explain at least 95% of the variance (the mean explained variance across all conditions for all participants with the first 8 principal components was 95.49% ± 0.83).

To evaluate the degree of feedback or feedforward control during movement execution in the different conditions, we measured the trajectory tangling (Russo et al., 2018; Perich et al., 2020). Tangling analysis is based on the principle that in dynamic systems the current state of the system strongly influences the future state. Recent studies (Russo et al., 2018; Perich et al., 2020) suggest that a fully feedforward trajectory will have low tangling because the current activity deterministically predicts future activity, whereas high tangling indicates a system that is driven by unexpected inputs, hence more feedback driven (see Figure 1 of Perich et al., 2020).

For the tangling analysis we used the first 8 principal components obtained from the PCA analysis on the kinematic data. The behavioral state $x_{t}$ is the matrix containing scores of trials projected onto the first three principal components, while ${\dot{x}}_{t}$ represents the velocity of the behavioral state. Tangling index was computed as follow (Russo et al., 2018):


$$Q\left(t\right)=\max_{t\prime }\frac{{\left\|{\dot{x}}_{t}-{\dot{x}}_{t\prime }\right\|}^{2}}{{\left\|x_{t}-x_{t\prime }\right\|}^{2}+\varepsilon }$$

In the equation the Euclidean Norm squared distance between derivatives to the numerator was employed, and the Euclidean Norm squared distance between states to the denominator plus a constant ε, calculated as in Russo et al. (2018). The metric looks over all time points for all trials for each position and is maximal when a similar position in state space ( x ), which gives a small denominator value, corresponds with different state space velocities ( $\dot{x}$ ), which gives a large value in the numerator.

We computed the tangling independently for all trials for each condition. Since the trials had different lengths, to compute the average tangling index over time, we divided the tangling data of each trial into 10 equal consecutive bins, each bin comprising data from 10% of the movement time.

The analysis of loadings was performed by deriving the weight of each individual kinematic variable from the main components of the PCA. We considered the absolute value of the weights of the variables and summed them in the first 8 PCs. Thus, we obtained information regarding the weights of individual variables according to the experimental condition we wanted to observe.

The statistical reliability of differences between mean reaction times, movement times, precision, accuracy, and tangling index was tested using between-subjects repeated-measures analysis of variance (ANOVA, *p* < 0.05) with handedness (right-handed and left-handed) as a between-participants factor.

To measure the effectiveness of the cue in directing attention, the differences in reaction times were evaluated with a 2-way repeated measures ANOVA with Arm (dominant and non-dominant) and Type of trial (reach to attended location, reach to unattended location, reach without constrained attention-valid, reach without constrained attention-invalid) as within-participants factors.

To evaluate hemispatial effects and whether they were influenced by the direction of attention, we evaluated the differences in reaction times, movement times, precision, and accuracy by performing two 2-way repeated measures ANOVAs for each variable with the following within-participants factors: Reaching side (ipsilateral, i.e., reaching toward the same side as the hand used, and contralateral, i.e., reaching toward the side opposite to the hand used), Direction of attention (right, not constrained, left). The differences in tangling index were evaluated with two 3-way repeated measures ANOVAs with Reaching side (ipsilateral, contralateral), Direction of attention (right, not constrained, left) and Temporal bin (from bin1 to bin10) as within-participants factors. We used only data recorded during valid trials (all MotorATN trials and valid Motor trials).

To evaluate the loadings of the kinematic variables according to the reaching side and attention direction, we performed a 4-way repeated measures ANOVA with the following within-participants factors: Reaching side (ipsilateral, contralateral), Direction of attention (right, not constrained, left), Effector (index, wrist), and Parameter (position, velocity, acceleration). We collapsed the loadings of the 3 coordinates x, y, and z of the index finger and wrist.

Whenever sphericity was violated (Mauchly test, *p* < 0.05), we used the Huynh-Feldt correction. All post-hocs were carried out using the Newman Keuls correction for multiple comparisons.

## Results

We evaluated the influence of the direction of spatial attention during reach planning on advantages in behavioral variables observed during the initiation and execution of ipsilateral arm reaching (hemispatial effects). To do this, we designed a task to direct spatial attention and motor planning to the same or different hemifields. Right-handers and left-handers performed reaching movements toward ipsilateral or contralateral targets using either the dominant or the non-dominant arm (see Methods). Reaction time data supported the effectiveness of the task design in directing attention and motor plan (see Supplementary Results, Supplementary Figure S1) and excluded consistent contribution of Simon effect (Supplementary Results). We evaluated the reaction times and motor outcomes and the dynamics of motor control when attention was constrained in different directions during ipsilateral and contralateral reaching.

### Hemispatial effects: reaction times

**Figure 2:**
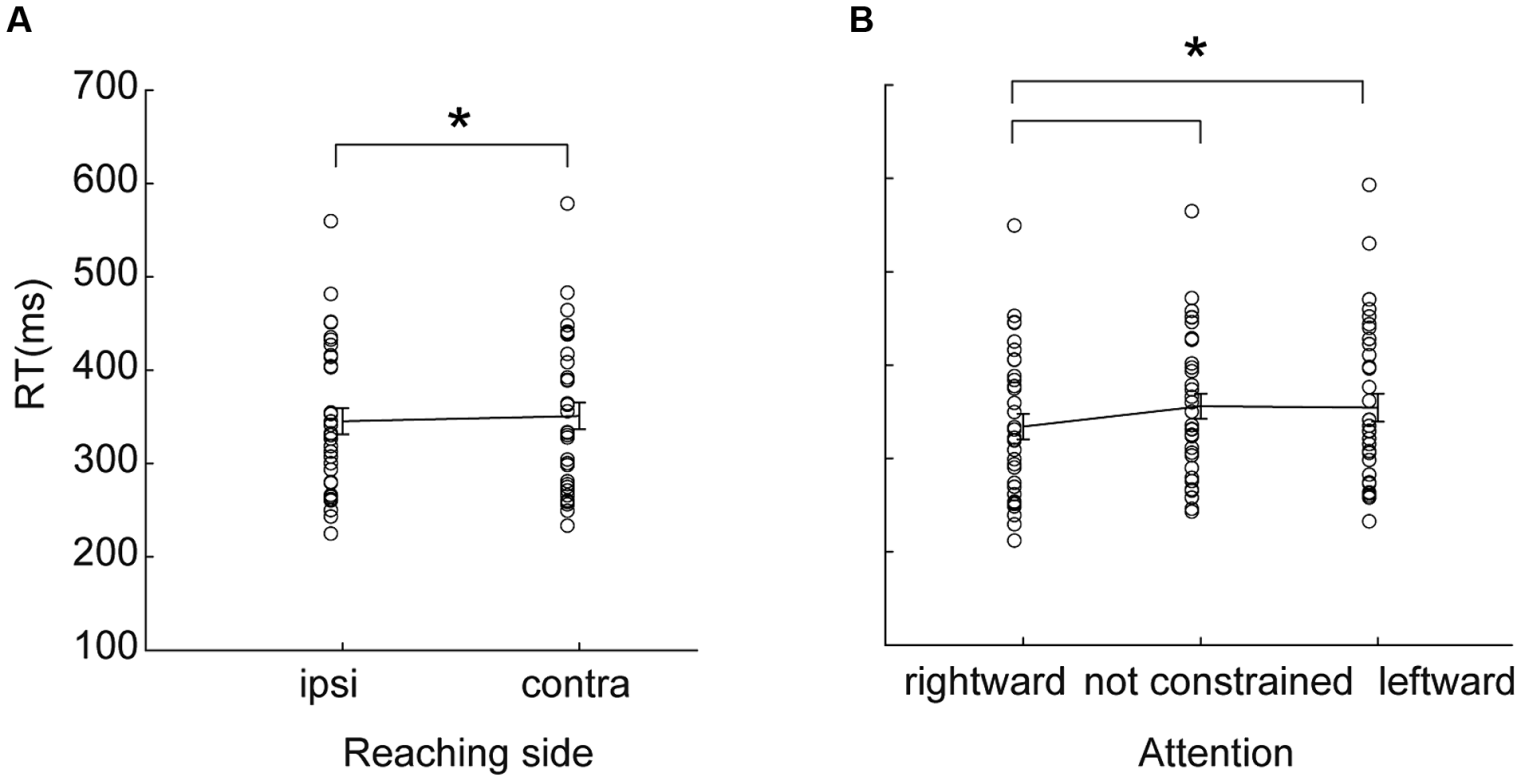
**(A)** Hemispatial effects on reaction times (RT) and effects of attention. Ipsi, ipsilateral reaching; Contra, contralateral reaching. **(B)** Effects of the direction of endogenous attention on reaction times. Rightward, attention directed rightward; Not constrained, endogenous attention not constrained; Leftward, attention directed leftward. Circles represent individual values. Error bars represent standard error (SE). Asterisks represent significant (*p* < 0.05) statistical comparisons.

To investigate the hemispatial effects on reaction times, we studied the influence of Reaching side and of Direction of attention on reaction times. Reaction times were modulated by the Reaching side [main effect, *F*_(1,32)_ = 4.83, partial eta squared = 0.13, *p* = 0.04, Figure 2A], in that reaction times during ipsilateral reaching were lower than during contralateral reaching. Moreover, we have found a main effect of the Direction of attention [*F*_(2,64)_ = 14.90, partial eta squared = 0.32, *p* < 0.001, Figure 2B] in that, when attending rightward, the reaching initiation was more prompt (all *p* < 0.001) than when attending leftward or when endogenous attention was not constrained, regardless of the reaching side. The reaction times of trials where attention was directed leftward were not dissimilar to reaction times of trials where attention was not constrained (*p* = 0.73). The interaction Reaching side by Direction of attention was not significant [*F*_(2,64)_ = 2.21, partial eta squared = 0.001, *p* = 0.96]. No effects of handedness have been found (all *F* < 2.21, all *p* > 0.11, all partial eta squared <0.06).

### Hemispatial effects: movement time

**Figure 3:**
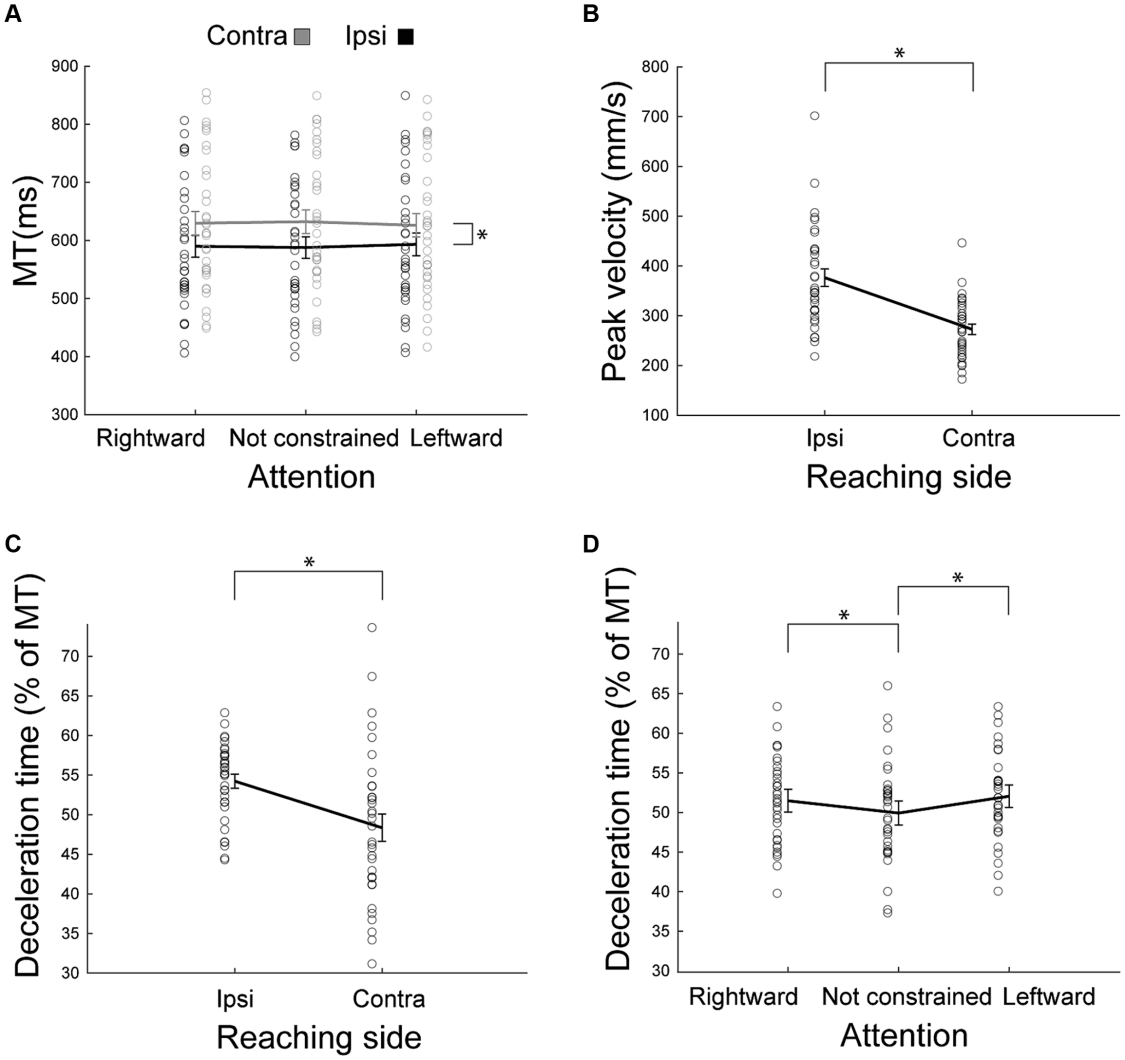
Behavioral parameters as outcomes of reaching. **(A)** Mean movement time as a function of the reaching side and of the direction of attention. Ipsilateral (ipsi) movements were faster than contralateral (contra) ones. The effect of the direction of attention was not supported by posthoc analysis. **(B)** Peak velocity as a function of reaching side. Ipsilateral peak velocity was higher than that of the contralateral movements. **(C,D)** Deceleration time, expressed as the percentage of movement time after peak velocity as a function of the reaching side **(C)** or of the direction of attention **(D)**. Other conventions as in Figure 2.

In agreement with the results found by Carey et al. (1996), we found here that movement durations provide very rich information regarding the influence of hemispace on movement (Figure 3A). We found a significant Reaching side by Direction of attention interaction effect [*F*_(2,62)_ = 4.33, *p* = 0.02, partial eta squared = 0.12, Table 1], likely driven only by the longer time spent during contralateral reaching (all *p* < 0.0002), because the effect of direction of attention was not supported by post hoc comparisons (all *p* > 0.09). Movement time was not dissimilar in right and left handers (all *F* < 2.16, all partial eta squared<0.06, all *p* > 0.13). In summary, these data suggest that ipsilateral reaching has a movement duration advantage (hemispatial effect) and that this effect was neither influenced by the direction of attention nor by handedness.

**Table 1:** Mean movement times (MT) across the different reaching sides and attention.

	MT (ms)	SE
Ipsilateral, attention to the right	591.37	18.81
Ipsilateral, attention not constrained	588.79	18.62
Ipsilateral, attention to the left	594.75	19.33
Contralateral, attention to the right	631.41	19.88
Contralateral, attention not constrained	633.73	20.16
Contralateral, attention to the left	627.66	19.81

SE, standard error.

### Hemispatial effects: peak velocity and deceleration time

We found that the peak velocity is lower in contralateral reaching [*F*_(1,32)_ = 40.23, partial eta squared = 0.56, *p* < 0.001, Figure 3B]. This effect was regardless of handedness (all *F* < 0.46, all partial eta squared<0.03, all *p* > 0.32). The direction of attention was not effective in modulating peak velocity (all *F* < 1.14, all partial eta squared <0.03, all *p* > 0.32). The interaction Reaching side by Direction of attention was not significant [*F*_(2,64)_ = 0.01, partial eta squared = 0.0004, *p* = 0.99].

Participants spent more time decelerating during ipsilateral reaching (main effect of Side of Reaching *F*_(1,32)_ = 10.19, partial eta squared = 0.24, *p* = 0.003, Figure 3C). Furthermore, deceleration time was longer when attention was constrained [main effect of Direction of attention *F*_(2,64)_ = 9.61, partial eta squared = 0.23, *p* = 0.0002, Figure 3D] than when it was not constrained (all *p* < 0.005). We did not observe significant differences between deceleration times when attention was directed leftward or rightward (*p* = 0.25). The interaction Reaching side by Direction of attention was not significant [*F*_(2,64)_ = 3.00, partial eta squared = 0.09, *p* = 0.06]. All these effects were irrespective of handedness (all *F* < 1.50, all partial eta squared <0.04, all *p* > 0.22).

### Hemispatial effects: reaching precision and accuracy

Reaching precision was not affected either by the side of reaching (all *F* < 1.67, all partial eta squared <0.05, all *p* > 0.20), or by the direction of attention (all *F* < 2.99, all partial eta squared <0.08, all *p* > 0.06); neither was it affected by handedness (all *F* < 1.32, all partial eta squared <0.04, all *p* > 0.27).

Participants showed a similar horizontal accuracy in both hemispaces, without significant ipsilateral advantages. Neither the direction of attention nor the handedness (all *F* < 1.55, all partial eta squared <0.05, all *p* > 0.21) was effective in modulating accuracy.

### Interim summary

In summary, we observed hemispatial effects in reaction times, movement duration, peak velocity, and deceleration time that were not dissimilar in right- and left-handers. The prompter reach initiation, the higher velocity and shorter movement time found in ipsilateral reaching were accompanied by a longer deceleration time. Interestingly, the changes in kinematic parameters during movement did not have any corresponding changes in movement accuracy and precision.

The direction of attention did not differently modulate movement time or peak velocity. Rather, the increase in deceleration time when the direction of attention was constrained during planning may be indicative of a regime of movement control that is more influenced by feedback signals during movement execution. Moreover, the decrease of reaction times during rightward attention suggests a more ballistic control of reaching in this condition. To test the potential weight of feedback signals over the entire duration of the movement in conditions in which attention was constrained toward different directions, and to gain insights of how attention modulates movement control, we applied principal component analysis on index and wrist kinematics and quantified movement dynamics by calculating the tangling index over time (Russo et al., 2018; Perich et al., 2020). Results are as follows.

### Hemispatial effects: regime of the dynamical system underlying movement

**Figure 4:**
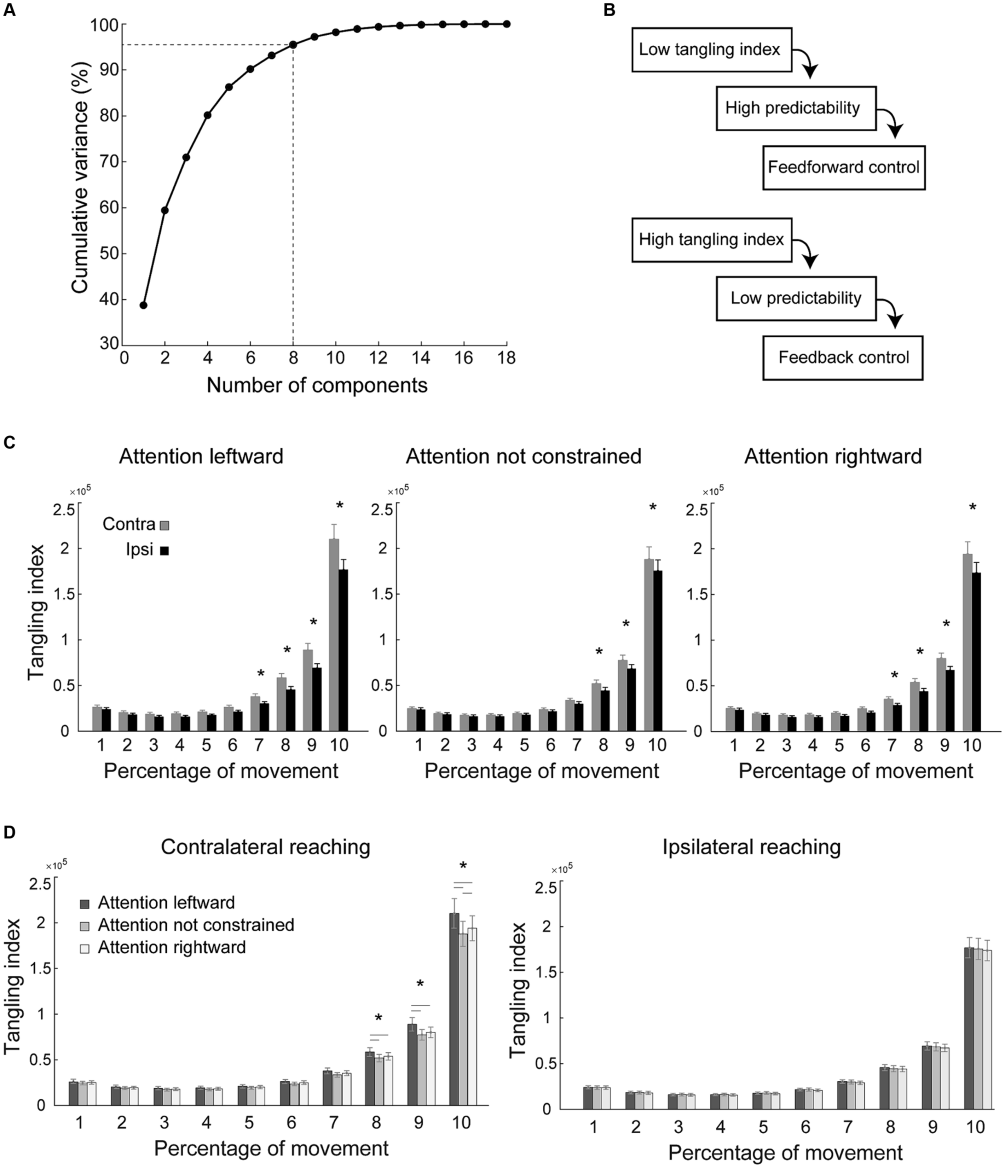
Principal component analysis and tangling index. **(A)** Principal component analysis: proportion of variance explained by the principal components. With 8 principal components more than 95% of the variance is explained. **(B)** Influence of unexpected inputs measured on behavioral trajectories. Tangling index quantifies the regime of a dynamical system (low tangling: feedforward dynamics; high tangling: feedback-driven dynamics). **(C,D)** Analysis of tangling index: effects of the Time bin by Reaching side by Side of attention interaction on the tangling index, indicative of a different motor control during contralateral compared to during ipsilateral reaching **(C)** and, during contralateral reaching, when attention was directed leftward compared to when attention was directed rightward, or when it was not constrained **(D)**. Tangling indexes were divided into 10 time-bins, each comprising 10% of the movement time. Other conventions as in Figures 2, 3.

To evaluate the influence of the direction of spatial attention during reach planning on motor control during reaching execution, we performed principal component analysis and computed the tangling index over time (see Figures 4A,B, Methods, Russo et al., 2018). Tangling analysis is based on the principle that in dynamic systems the current state of the system strongly influences the future state. A fully feedforward trajectory will have low tangling because the current activity deterministically predicts future activity, whereas high tangling indicates a system that is driven by unexpected inputs, hence by feedback (see Figure 4B and Figure 1 of Perich et al., 2020). Tangling index is thus considered a measure of how much motor control is driven by feedforward or by sensory feedback (Russo et al., 2018; Perich et al., 2020).

We found a significant interaction between Reaching side, Direction of Attention and Time bin, [*F*_(18,576)_ = 3.16, *p* < 0.001, Mauchly test *p* < 0.001, Huynh-Feldt adjusted *p* = 0.04, partial eta squared = 0.09, Figures 4C,D]. We found hemispatial effects on tangling index in the form of a higher tangling index (suggestive of a motor control that is more feedback-driven) in contralateral reaching than in ipsilateral reaching (see asterisks in Figure 4C). These effects were observed in the last phases of the movement when attention was directed to the right (Figure 4C, right; bin 7–10 all *p* < 0.005, the remaining bins *p* > 0.35), when attention was not constrained (Figure 4C, middle; bin 8–10, all *p* < 0.005, the remaining bins *p* > 0.27), and when attention was directed leftward (Figure 4C, left; bin 7–10, all *p* < 0.005, the remaining bins all *p* > 0.22). The regime of the dynamical system underlying ipsilateral reaching was not influenced by the direction of attention (all *p* > 0.35, Figure 4D right). Instead, during contralateral reaching, the direction of attention was effective in changing the regime of motor control (see asterisk in Figure 4D, left): when attention was constrained leftward, the last 30% of the movement was more feedback-driven than when attention was constrained rightward (bin 8–10, all *p* < 0.001; all the remaining bins *p* > 0.17) or not constrained (bin 8–10, all *p* < 0.04; all the remaining bins *p* > 0.81), conditions that in turn were not statistically different in any of the bins (all *p* > 0.08), except for the last one (*p* < 0.001). None of the above-mentioned effects depended on handedness (all *p* > 0.08).

In summary, attention constrained leftward during planning of contralateral reaching exerted a long-term effect on motor control during reaching execution in that, in the last phases of the movement, it showed a higher reliance on feedback signals than when attention was not constrained or was directed rightward.

### Analysis of variable loadings

**Figure 5:**
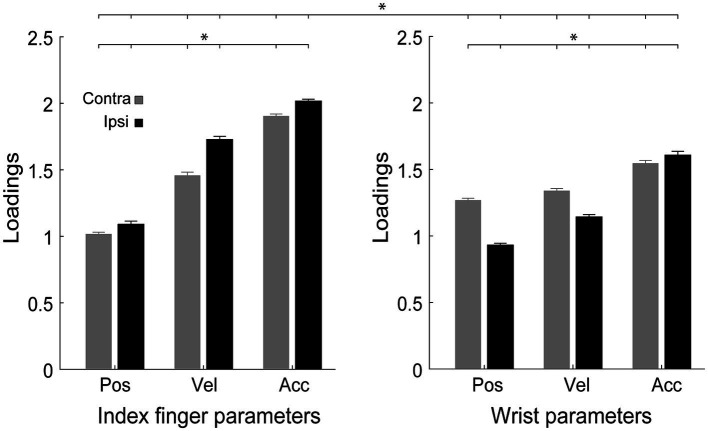
Analysis of the loadings of the variables in the PCA. Distribution of loadings of the parameters (pos, position; vel, velocity; acc, acceleration) for each effector (index finger, wrist), during contralateral and ipsilateral reaching. Other conventions as in Figures 2–4.

Velocity, position, and acceleration of the wrist and index finger had variable weights in the PCA, indicating that they were differently relevant in the explanation of the observed variability. We found a significant effect of the interaction of Reaching side, Effector and Parameter on loadings [*F*_2,58_ = 126.1, *p* < 0.0001, partial eta squared = 0.8, Figure 5], irrespective of handedness (all *F* < 1.00, all partial eta squared <0.03, all *p* > 0.37) and of the direction of attention (all *F* < 1.8, all partial eta squared <0.05, all *p* > 0.12). Regardless of the effector, acceleration was the most relevant parameter to explain the variability (all *p* < 0.001). During ipsilateral reaching, the index finger parameters were always more relevant than the wrist parameters (all *p* < 0.001), whereas during contralateral reaching this trend was observed only for velocity and acceleration (all *p* < 0.001). Moreover, the index finger variables were more relevant in ipsilateral than in contralateral movements, whereas the wrist variables showed the opposite trend except for acceleration (all *p* < 0.001).

## Discussion

We investigated hemispatial effects and then whether spatial attention during reach planning modulates reaching parameters like reaction time, movement time, accuracy, precision, peak velocity, movement deceleration, and movement dynamics. We used a task that was already shown to be effective in controlling attention and movement plan independently (Messinger et al., 2021). Reaction times analysis of our data confirmed that the cue was able to spatially dissociate direction of attention and movement plan (Supplementary Figure S1), and that no consistent Simon effects were found.

In agreement with other studies (Berlucchi et al., 1971; Van Der Staak, 1975; Prablanc et al., 1979; Di Stefano et al., 1980; Bashore, 1981; Fisk and Goodale, 1985; Carson et al., 1990, 1992, 1993; Marzi et al., 1991; Chua and Elliott, 1993; Hoptman and Davidson, 1994; Ingum and Bjørklund, 1994; Carey et al., 1996; Levin, 1996), reaction time, movement time, peak velocity, and deceleration time were modulated by the reaching side. Contralateral movements showed longer reaction times and movement times, lower peak velocity, and shorter deceleration time (Figures 2A, 3A–C) with respect to ipsilateral reaching. By exploring the dynamics of ipsilateral and contralateral movements, we also found that they had different tangling index values, suggestive of a different motor control (see black asterisks in Figure 4C). Specifically, during ipsilateral reaching, the lower tangling indexes suggest a high degree of predictability of future states, which is suggestive of a feedforward or autonomous dynamical system, as suggested also in previous studies (Russo et al., 2018; Perich et al., 2020). Contrarily, during contralateral reaching, we found higher tangling indexes, that indicate a system more likely driven by unexpected inputs [i.e., more feedback-driven (Russo et al., 2018; Perich et al., 2020)]. This can explain the longer reaction and movement time, the lower peak velocity, and the shorter deceleration time found in contralateral reaching. This could represent a mechanism aimed at adjusting the precision and accuracy in a more disadvantaged condition (contralateral reaching) to obtain the same precision and accuracy found for the more favorable ipsilateral reaching. Indeed, precision and accuracy were not modulated by reaching side.

We show here that during movement execution there is an increasing trend of tangling indexes (Figures 4C,D) while the ipsilateral or contralateral movements unfold. The increase in tangling index with movement progression suggests an increase in uncertainty in motor control (Russo et al., 2018; Perich et al., 2020). According to the optimal feedback control framework (Todorov and Jordan, 2002; Liu and Todorov, 2007; Franklin et al., 2012; Dimitriou et al., 2013; Voudouris and Fiehler, 2021), visuomotor and somatosensory gain increase midway through the movement and decrease as the hand approaches the target, leaving the lead to the motor-related feedback input that is needed to stop the movement rather than sensory gain controlling hand position (Dimitriou et al., 2013). The increase in tangling indexes toward the end of movement reflects these ‘stop-signals’ and the uncertainty that is due to the reduction of the visuomotor and somatosensory gains and shows that this process is more powerful during contralateral reaching.

Attention modulated reach dynamics. As hypothesized (Bowers and Heilman, 1980; Heilman and Van Den Abell, 1980; Reuter-Lorenz et al., 1990; Benwell et al., 2014), we did observe a left visual field bias in the dynamics of motor control of contralateral reaching, but it followed a specific time course. When attention was directed leftward during reach planning, we found higher tangling indexes in the last 30% of the movement time, compared to when attention was not constrained or was directed to the right. Motor control during contralateral reaching has a higher degree of uncertainty, as indicated by higher tangling indexes compared to the ipsilateral condition. Directing spatial attention leftward makes the system even more uncertain, but probably more capable to consider feedback-related input regarding movement damping, given the time course of this effect that is restricted during the last parts of the movement, and the necessity to deal with the disadvantages of reaching toward contralateral targets.

The PCA analysis revealed that acceleration of either the index finger or the wrist was the most relevant parameter with which to explain the variance (Figure 5). This is in agreement with many studies that have found that acceleration is the most difficult kinematic parameter to estimate (Gottsdanker et al., 1961; Calderone and Kaiser, 1989; Brouwer et al., 2002; Watamaniuk and Heinen, 2003), probably because visual motion related neurons accurately encode target velocity and direction, but have only partial information regarding acceleration (Perrone and Thiele, 2001), or because the timing of the encoding of acceleration is not compatible with the sensorimotor delays that are typical of motor control judgments (Tresilian, 1995). Moreover, variables taken from the index finger are generally more relevant than those relative to the wrist in explaining variance. This is likely due to the higher number of degrees of freedom in the index finger than in the wrist and suggests the greater relevance of the index finger in a reach-to-point task such as ours.

While there are many studies with right-handed participants, studies with left-handers are relatively scarce. Nevertheless, it is widely accepted that only subtle differences are present in the motor-related domain between left-handers and right-handers: for example, left handers seem less lateralized than right handers in the control of movements executed with the ipsilateral hand (van den Berg et al., 2011), in motor skill learning or motor threshold (Triggs et al., 1994; McGrath and Kantak, 2016), and in motor cortex functions (Civardi et al., 2000); they also have a less lateralized network for action understanding (Kelly et al., 2015), in agreement with the handedness-dependent laterality of the motor system (Hiraoka et al., 2018). This weak lateralization may depend on a stronger interhemispheric connectivity of left-handers, given their larger corpus callosum (Witelson, 1985; Beaton, 1997; Luders et al., 2010). Our data suggest that the interactions between attention and movement are similar in left- and right-handers, using either hand, in accordance with the slight differences found in the brain organization of these two populations (Sha et al., 2021).

### Potential applications

Understanding the interactions between attention and motor planning can be crucial to improving motor control in patients. Several studies on motor control have focused on aspects directly related to the neuromuscular system, such as biomechanics or reflexes (Latash and Zatsiorsky, 2015) but more recently other cognitive factors, like attention, have been found to be fundamental for the performance of the movement (see Song, 2019 for a review). Therefore, understanding how attention influences motor planning could enable therapists to design targeted therapeutic interventions that enhance both functions. In the sensory domain, providing visual feedback during a motor task can increase the patient’s attention and improve movement accuracy and coordination. Moreover, motor strategies that emphasize focused attention on specific movement details could led to faster improvements. In the assistive devices domain, wearable devices or virtual reality systems can be designed to provide real-time feedback on attention and motor planning during the execution of specific motor tasks. In summary, understanding the interactions between attention and motor planning can open new opportunities to develop more effective therapeutic interventions and innovative assistive technologies to improve motor control in patients with motor disorders.

### Potential limitations of the study

In our task, only one target per hemifield was tested, limiting the possibility of investigating the effect of eccentricity on the results. Using more than one target would have significantly raised the number of trials of the experiment, which is currently quite high for such a demanding task. An increase of the total number of trials would have likely decreased the performance of the participants due to tiredness, and would have excessively increased the total time required to complete the experimental session with an increase in fatigue.

## Conclusion

Kinematic parameters such as reaction time, movement time, peak velocity, and deceleration time reflect advantages in ipsilateral reaching compared to contralateral one. The dynamics of motor control underlying reaching differ between hemifields, since motor control during contralateral reaching shows a higher degree of uncertainty which may be likely due to a higher influence of feedback signals. The difference in kinematic parameters and motor control partially compensate one to another so to obtain movements with similar precision and accuracy regardless of the side of reaching. We found asymmetries in the influence of attention in motor control, as leftward attention during motor planning makes the motor control more uncertain and thus likely more feedback-driven. These mechanisms are common in right- and left-handers.

## Data availability statement

The datasets presented in this article are not readily available because of privacy restrictions, but are available from the corresponding author upon reasonable request. Requests to access the datasets should be directed to RoB, rossella.breveglieri@unibo.it.

## Ethics statement

The studies involving humans were approved by Comitato di Bioetica, Università di Bologna. The studies were conducted in accordance with the local legislation and institutional requirements. The participants provided their written informed consent to participate in this study.

## Author contributions

RoB: Conceptualization, Data curation, Investigation, Methodology, Software, Supervision, Validation, Writing – original draft, Writing – review & editing. RiB: Investigation, Methodology, Software, Writing – review & editing. SD: Methodology, Software, Writing – review & editing. ML: Conceptualization, Funding acquisition, Methodology, Validation, Writing – review & editing. CG: Conceptualization, Methodology, Validation, Writing – review & editing. PF: Funding acquisition, Writing – review & editing.
