# Supplementary material for: Modulation of reaching by spatial attention
Source: Front Integr Neurosci. 2024 May 15;18:1393690. doi: 10.3389/fnint.2024.1393690 (PMC11138159; doi:10.3389/fnint.2024.1393690)
Supplement: Supplementary file 1 [file Data_Sheet_1.docx]

**Modulation of reaching by spatial attention.**

**Rossella Breveglieri^1^*, Riccardo Brandolani^1,2^, Stefano Diomedi^1^, Markus Lappe^3^, Claudio Galletti^1^, Patrizia Fattori^1^.**

**Supplementary methods**

To reveal potential Simon effect(Simon and Rudell, 1967; Hommel, 2011), we performed a 2-way repeated measures ANOVA on reaction times of MotorATN trials with the following within-participants factors: Cue side (right, left), Arm (right, left) and with handedness as a between-participants factor.

**Supplemental results**

*Effectiveness of our task design and potential influence of Simon effect.*

We tested whether attention was allocated as instructed by measuring reaction times to Go signal detection (Go, Fig. 1A). We wanted to support the effectiveness of the task in directing participants’ attention by verifying if reaction times during reaching to attended targets were shorter than during reaching to unattended targets, because planning a movement to a unattended position would imply a cost for the system. Also, reaction times in invalid trials would be longer than in valid trials, in agreement with Posner’s classical findings (Posner, 1980). The comparison of reaction times of different conditions confirmed these hypotheses. In fact, reaction time to detection of the Go signal was significantly modulated by the Type of trial (F_(3,96)_=31.18, p<0.001, partial eta squared=0.49, see Table S1 and Fig. S1) irrespectively of the arm used (all F<2.28, all partial eta squared < 0.06, all p> 0.14) or of handedness (all F<3.36, all partial eta squared < 0.10, all p> 0.08). Specifically, participants were faster in detecting the target when attention and motor plan were directed to the same side than when attention and movement planning were directed to opposite sides (p=0.006), or when the direction of spatial attention was not constrained (p=0.001, Fig. S1). Similar reaction times were observed when attention and motor plan were in opposite hemifields compared to when attention was not constrained in valid trials (p=0.44). The detection of targets for invalid trials was the slowest compared to all the other trials (all p=0.001). Although we recognize that there are no direct measures for attention, reaction time is a common indirect measure of attention (e.g. (Jonides and Gleitman, 1976; Jonides, 1980, 1983; Posner, 1980; Rizzolatti et al., 1987; Seya and Mori, 2012; Ciavarro et al., 2013; Messinger et al., 2021)), so all these data suggest that in our task, spatial attention and motor plan were directed as instructed. Moreover, the reaction time advantage of both right-handed and left-handed participants in target detection during reaching to attended targets supports the hypothesis that we are able to direct attention and motor plan to opposite hemispaces (Baldauf et al., 2006; Baldauf and Deubel, 2010; Schonard et al., 2022).

In the experimental design, the Go cue is spatially cued (left or right part of a central square). One could argue that this spatial cueing causes an additional spatial in/compatibility effect, known as the Simon effect (Simon and Rudell, 1967; Hommel, 2011), that could lead to faster responses of the right hand when the colored square is located right of the fixation point (spatially compatible conditions) than when it is located left of the fixation point (spatially incompatible conditions). The result pattern should reverse for left arm reaches. To examine the potential contribution of the Simon effect in the current data, we rearranged the reaction times to test their advantages for spatially compatible trials. It can be hypothesized that Simon effects could be revealed by a significant interaction Cue Side by Arm. On the contrary, this interaction did not significantly (p=0.25) affect the reaction times. Indeed, we only find that reaction times were affected by the interaction Cue Side by Arm by Handedness (F_(1,32)_=4.44, partial eta squared=0.12, p=0.04), suggesting different effects in right- and left-handers. Specifically, we found even more complex effects, in that the Simon effect was found only when movements were performed with the right arm in both groups: reaction times were lower in spatially compatible than in spatially incompatible trials in right handers (p=0.001) and in left handers (p=0.02), both using the right hand. With the left hand, no advantage for spatially compatible trials was found (p=0.46) in right handers, whereas in left handers the spatial compatibility was even a disadvantage (p=0.002). Thus, no consistent Simon effect was found and therefore we suggest that this could not be considered as a potential confound in the data interpretation.

We didn’t find any Simon effects in motor-related parameters (Table S2).

**Table S1**

**Mean reaction times (RT) across the different Types of Trials. SE=standard error.**

| Type of trial | Mean RT (ms) | SE |
| --- | --- | --- |
| MotorATN cued congruent | 338.30 | 13.47 |
| MotorATN cued incongruent | 353.50 | 13.82 |
| Motor cued valid | 357.17 | 13.09 |
| Motor cued invalid | 388.71 | 13.57 |

**Table S2**

**Absence of Simon effects on movement-related parameters.**

| **Parameter** | **All F** | **All partial eta squared** | **All p** |
| --- | --- | --- | --- |
| Movement time | <0.04 | < 0.001 | >0.84 |
| Peak velocity | <1.27 | <0.04 | >0.27 |
| Deceleration time | <2.80 | <0.09 | >0.08 |
| Reach precision | <0.73 | <0.02 | >0.40 |
| Reach accuracy | <0.13 | <0.01 | >0.51 |
| Tangling index | <1.07 | <0.03 | >0.38 |

*
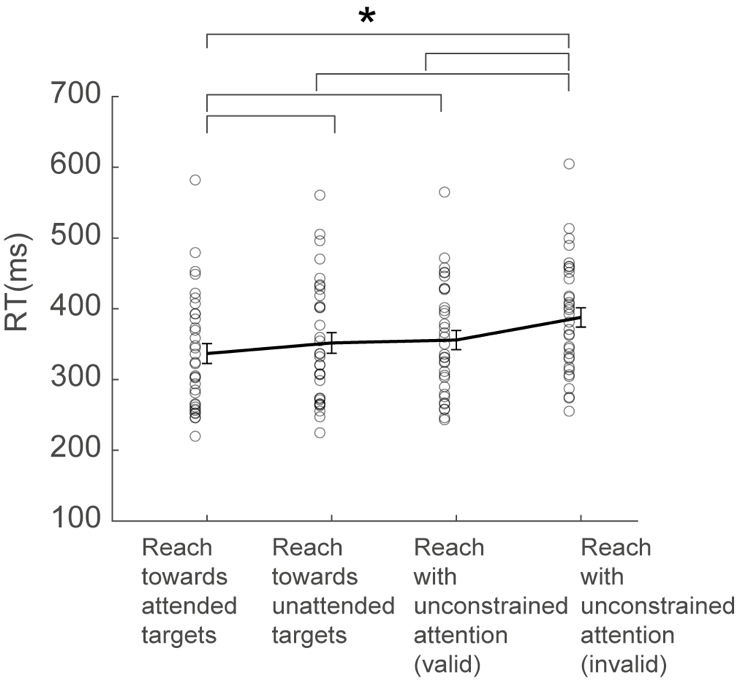
*

Figure S1

Mean reaction times as a function of the Type of trial, when reaching was performed toward attended locations, unattended ones, and without constraining of endogenous attention. Circles represent individual values. Error bars represent standard error (SE). Asterisks represent significant (p<0.05) statistical comparisons.

**Supplementary references**

Baldauf, D., and Deubel, H. (2010). Attentional landscapes in reaching and grasping. *Vision Res* 50, 999–1013. doi: 10.1016/j.visres.2010.02.008

Baldauf, D., Wolf, M., and Deubel, H. (2006). Deployment of visual attention before sequences of goal-directed hand movements. *Vision Res* 46, 4355–74. doi: 10.1016/j.visres.2006.08.021

Ciavarro, M., Ambrosini, E., Tosoni, A., Committeri, G., Fattori, P., and Galletti, C. (2013). rTMS of Medial Parieto-occipital Cortex Interferes with Attentional Reorienting during Attention and Reaching Tasks. *J Cogn Neurosci*, 1–10. doi: 10.1162/jocn

Hommel, B. (2011). The Simon effect as tool and heuristic. *Acta Psychol (Amst)* 136, 189–202. doi: 10.1016/j.actpsy.2010.04.011

Jonides, J. (1980). Towards a model of the mind’s eye’s movement. *Canadian Journal of Psychology / Revue canadienne de psychologie* 34, 103–112. doi: 10.1037/h0081031

Jonides, J. (1983). Further toward a model of the Mind’s eye’s movement. *Bull Psychon Soc* 21, 247–250. doi: 10.3758/BF03334699

Jonides, J., and Gleitman, H. (1976). The benefit of categorization in visual search: Target location without identification. *Percept Psychophys* 20, 289–298. doi: 10.3758/BF03199455

Messinger, A., Cirillo, R., Wise, S. P., and Genovesio, A. (2021). Separable neuronal contributions to covertly attended locations and movement goals in macaque frontal cortex. *Sci Adv* 7. doi: 10.1126/sciadv.abe0716

Posner, M. I. (1980). Orienting of attention. *The Quarterly Journal of Experimental Psychology, 32(1), 3–25.* 32, 3–25. Available at: https://doi.org/10.1080/00335558008248231

Rizzolatti, G., Riggio, L., Dascola, I., and Umiltá, C. (1987). Reorienting attention across the horizontal and vertical meridians: Evidence in favor of a premotor theory of attention. *Neuropsychologia* 25, 31–40. doi: 10.1016/0028-3932(87)90041-8

Schonard, C., Heed, T., and Seegelke, C. (2022). Allocation of Visuospatial Attention Indexes Evidence Accumulation for Reach  Decisions. *eNeuro* 9. doi: 10.1523/ENEURO.0313-22.2022

Seya, Y., and Mori, S. (2012). Spatial attention and reaction times during smooth pursuit eye movement. *Atten Percept Psychophys* 74, 493–509. doi: 10.3758/s13414-011-0247-y

Simon, J. R., and Rudell, A. P. (1967). Auditory S-R compatibility: the effect of an irrelevant cue on information processing. *J Appl Psychol* 51, 300–4. doi: 10.1037/h0020586
